# Community-academic partnerships to embrace and ensure diversity, equity, and inclusion in translational science: Evidence of successful community engagement

**DOI:** 10.1017/cts.2023.601

**Published:** 2023-07-28

**Authors:** Joy P. Nanda, Roger S. Clark, Jennifer Ayana Harrison, Pamela Ouyang, Cyd Lacanienta, Cheryl Himmelfarb, The CRAC Editorial Contributors

**Affiliations:** 1 Community Research Advisory Council, Johns Hopkins Institute of Clinical and Translational Research, Baltimore, MD, USA; 2 Johns Hopkins University, Baltimore, MD, USA

**Keywords:** Community-academic partnership (CAP), diversity, equity, inclusion (DEI)

## Abstract

Community-Research Advisory Councils (C-RAC) provide a unique mechanism for building sustainable community-academic partnership, fostering bidirectional understanding of complex research issues, disseminating timely research findings, and thereby improving public trust in science. Created in 2009, the Johns Hopkins C-RAC has a mission to achieve diversity, equity, and inclusion (DEI) of stakeholders across the entire research continuum. It has nurtured over a decade of partnership among community and academic stakeholders toward addressing health disparity, health equity, structural racism, and discrimination. Evidence of successful strategies to ensure DEI in partnership and lessons learned are illustrated in this special communication.

## Introduction

Embracing multicultural diversity, equity in healthcare, and inclusion of community voices in healthcare settings are critical steps toward understanding and addressing healthcare needs of vulnerable and marginalized populations[1–4]. To this end, there is a growing appreciation of including the community voice in all phases of the research processes toward improving healthcare services[5–6]. Collective voices from a culturally diverse community can 1) alleviate negative perceptions about healthcare research, 2) effectively discuss health inequities in marginalized patients, 3) address structural racism and discrimination in healthcare, and 4) promote trust in the research process [7–9].

While the practice of cultural diversity, equity, and inclusion (DEI) is abundantly endorsed in biomedical, healthcare, and academic medical teaching settings [10–22], evidence of stepwise implementation of these endorsements is not extensively evaluated or documented [23–25].

To evaluate DEI practices, steps toward achieving a unified DEI that supports a sustainable community-academic partnership (CAP) need further documentation.

The overarching goal of the Community Research Advisory Council (C-RAC) of the Johns Hopkins Institute for Clinical and Translational Research (ICTR) has been to build a trustworthy relationship [26] among community members and academic researchers through education, and service activities. Specific objectives of the C-RAC have been to develop, implement, and monitor health services research-related activities that benefit both the academic and community partners indirectly or directly within the Greater Baltimore-Washington region.

Since its inception, the C-RAC has striven to build a sustainable community-academic partnership toward promoting successful community-engaged research (CER) and dissemination of research findings. Accordingly, this report illustrates DEI strategies and practices that have enhanced and sustained CER among Johns Hopkins researchers.

The concepts and constructs presented in this special communication are highlights of DEI activities as a result of successful community-academic partnership in healthcare research over the years. Several constructs presented here are in the process of being structured and operationalized for future reports.

## Materials and Methods

### Ongoing Community-academic Partnership Activities on DEI

The C-RAC was created in 2009 to provide a space where co-learning can take place between researchers and the community being served. Over its 14 years of existence, the C-RAC has evolved into a model fostering DEI in an insular, academic research setting. With bylaws on governance structure and subcommittees run by community partners, a working model was established to help shift the power dynamic between the research institute and the community with community partners seen as trusted experts based on lived experiences and environments in which they reside. Specific activities of the C-RAC include: (1) ensuring cultural diversity in race/ethnic, age, gender, sexual orientation, and occupational backgrounds in the C-RAC membership composition, (2) facilitating equitable access to healthcare research to address health disparities and social justice, (3) improving community awareness, trust, knowledge, understanding, and inclusion in all phases of the research continuum in relevant community-engaged research, and (4) documenting the engagement steps and processes to evaluate researcher commitment to a fully engaged community within the entire research process. These steps are routinely monitored by the community partners of the C-RAC with a unified approach and goal toward improving health outcomes and reducing health disparities.

### Conceptualizing and Operationalizing Trust

Since its inception, the C-RAC has adhered to seven operationalized [26,27] domains of trust as integral to DEI. These multi-dimensional domains included (1) acknowledging and working to address vulnerability among the community partners to fully understand research, (2) encouraging academic partners to fully explain the nuances and critical phases of the research continuum, (3) enhancing the belief that community partners will act and follow a set of guided principles of research, (4) sharing on values, visions, and goals toward trust-promoting plans and activities, (5) facilitating a set of collaborative tasks based on perceived skill set, expertise, and experience of each partner, (6) ensuring reliability and reciprocity of timely research tasks performed by both partners; and (7) power sharing and co-ownership of partnership activities. Steps and processes are currently in progress toward developing structured surveys on trust dimensions with the aims of psychometric testing, analysis and dissemination of findings in relation to community-engaged research.

**Figure 1.**
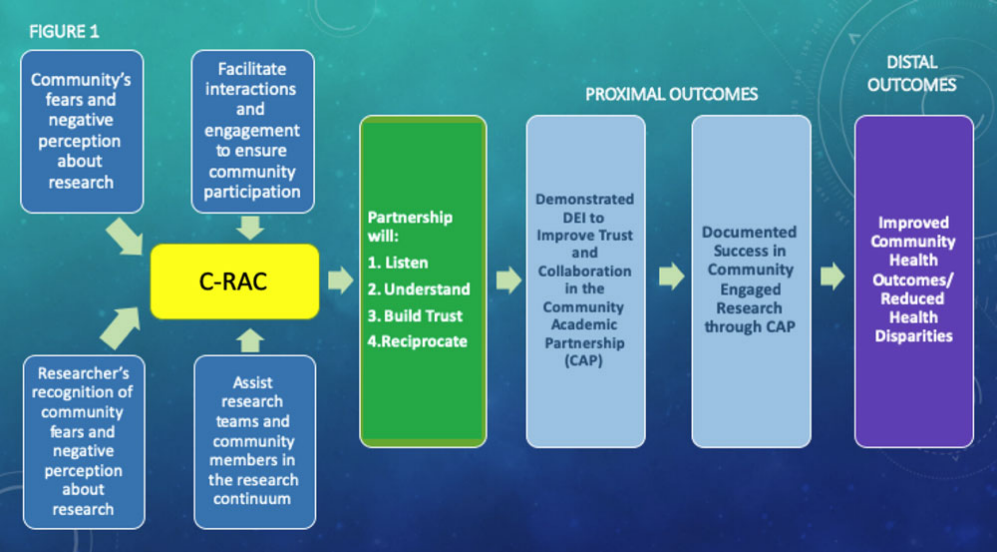
Unifying framework toward a sustainable community-academic partnership in research. C-RAC, Community Research Advisory Council.

The conceptual framework (Fig. 1) illustrates a unifying model for a successful community-academic partnership in research. A key feature in this framework is a sincere recognition by the academic partners of the mistrust and the negative perception about health services research and delivery that exist in the community. The unifying model promotes bidirectional engagement to build sustainable trust and ensure DEI for critical community issues. Using DEI strategies, the C-RAC translates community inputs to assist academic researchers throughout the research continuum and vice versa. These iterative processes facilitate both community and academic partners to listen, understand, and reciprocate concerns of both sides toward building a successful community-engaged research. Consequently, these iterative interactions and engagements generate a sustainable trustworthy community-academic partnership among researchers who solicit community inputs to succeed in their research endeavors (Fig 1).

**Figure 2.**
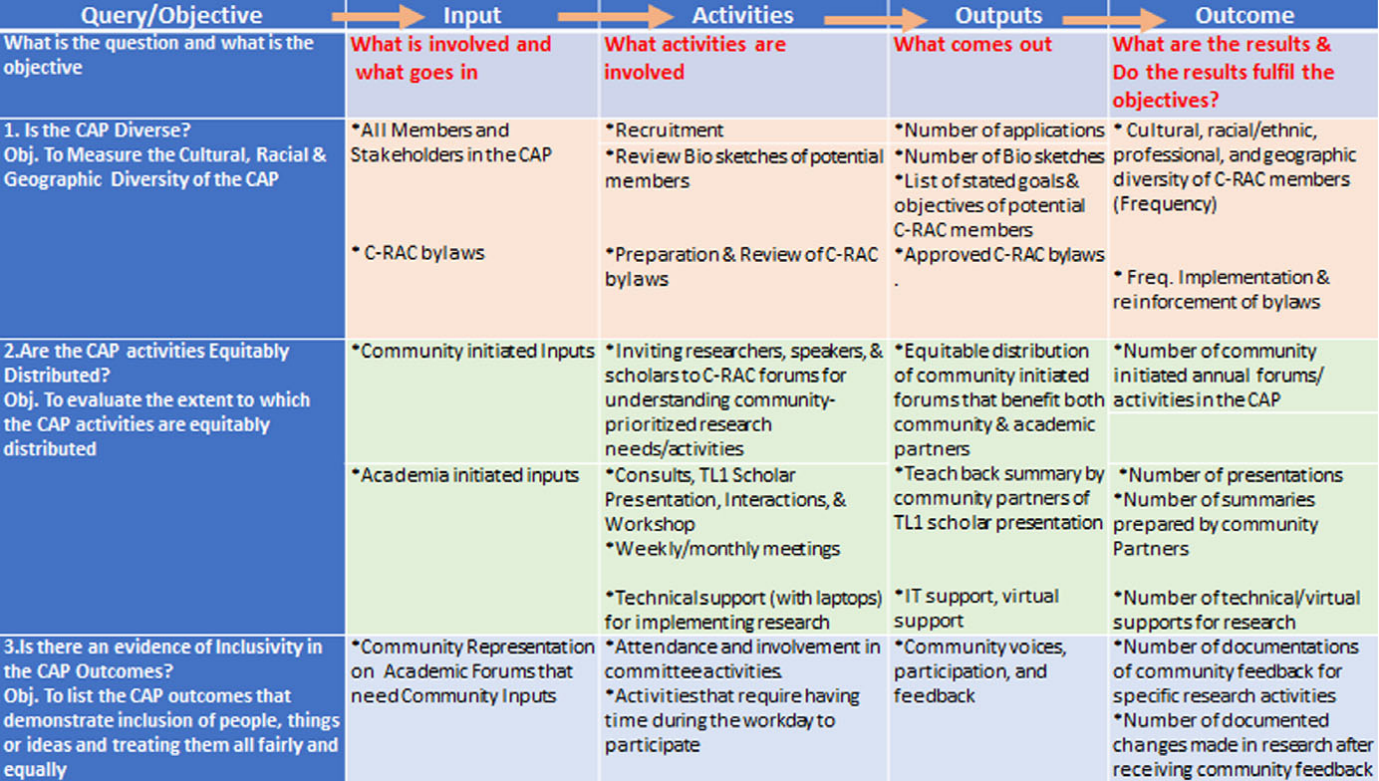
Logic model for DEI practices in the community-academic partnership. CAP, community-academic partnership; C-RAC, community- research advisory council; TL1, predoctoral training program.

The C-RAC’s activities adhere to structural and logical steps within the unifying DEI model. As an instructive road map to illustrate the resources or inputs required to achieve the DEI goals, the logic model in Fig. 2 depicts the step-by-step relationship among resources, planned work, and intended DEI results. The logic model also guides processes to evaluate desired goals and objectives. The logic model describes how inputs and activities based on DEI-relevant queries and objectives will lead to intended DEI outcomes.

The inputs, activities, and outputs circumscribe a unifying approach to DEI within the C-RAC and follow outcomes that address a specific inquiry or objective. For example, to evaluate that the C-RAC has a culturally and racially diverse membership, its composition is periodically recalibrated. Furthermore, to ensure equity in community voices in the research process, community leaders are invited to participate in ad hoc forums to identify and prioritize unmet health needs, thus paving the way toward future research endeavors. Similarly, the extent to which researchers reciprocate community feedback and document revisions in their research protocol has been a yardstick to demonstrate the inclusion of community voices toward achieving DEI goals in the CAP.

### Outcomes of DEI Efforts

The C-RAC strives to function as a DEI team within the community-academic partnership. Each DEI activity has shown promise that community voices can be heard toward alleviating the mistrust in the research continuum.

### Diversity

Per bylaws, C-RAC membership constitutes more than half of nonacademic, community partners. Cultural and geographic diversity (five counties) of the thirty-two members reflect five age groups (30–83 years), six race/ethnic groups (twenty-one Blacks/African Americans, five Whites, two Latinas, three Asians, one African), two-third females, three LGBTQ individuals, employed, retirees, and semi-retirees, community health navigators, activists, federal, state, and local administrators, academic support staff and faculty. Additionally, the C-RAC membership represents diverse stakeholder groups, e.g. community leaders, research participants, patients, lawyers, faith-based and business community, students, staff, and faculty. Overall, membership included seventeen community partners and fifteen faculty and staff from Johns Hopkins, herein termed as academic partners. On an average, each member attended sixteen out of the twenty-two scheduled meetings in a calendar year, served as member for at least 2 years, and actively participated in at least one subcommittee. Almost three-fourth (23 of 32) of C-RAC members have been actively participating in activities for 8–10 years.

### Equity

The C-RAC is engaged in three major efforts throughout the year: (1) *research reviews as* consultative services from community partners who provide guidance/advice to research teams throughout the grant writing and post-award research processes, (2) *community outreach* through two annual programs to provide cutting-edge information on prevention, treatment, research, and ethics of community prioritized clinical topics, and (3) availability of clinicians and researchers at local markets to discuss medical and research topics of interest to community members. To this end, the inputs of both the community and academic partners are equitably distributed in order to achieve measurable success of the aforementioned efforts. Overall, the academic partners have been very supportive of C-RAC with a goal to ensure that benefits of the collaborative knowledge-sharing activities flow equitably between community and academic partners.

Another example of equity in power sharing in the CAP is the composition of three (research and training; governance; community outreach) committees, each with a chair (elected by the C-RAC members) from community partners. A major task of each committee is to ensure DEI in C-RAC matters. Currently, ten community partners and three academic partners constitute the governance committee.

In matters of equity in CAP, community partners needed more direct, clear, and articulated communication with the academic partners. Community partners frequently indicated that they needed to clearly and comprehensively understand what researchers discussed on a particular topic. Thereupon, the “Teach Back” was a critical process on the part of both the community and academic partners that ensured discussion issues remained understandable, iterative, comprehensive, and transparent. The Teach-Back methodology, adapted from the Agency for Healthcare Research and Quality’s Health Literacy Universal Precaution Toolkit [28], encompasses several steps practiced by community partners in response to community consultations requested by the TL 1 research scholars. The TL 1 research scholar program is a competitive predoctoral scholarship as part of the CTSA to increase the translational research workforce and enhance career development of future leaders of the biomedical research workforce. The C-RAC’s teach-back process begins with one community partner (1) being assigned to the TL 1 research scholar who had requested C-RAC consultation services, as part of triaging, (2) having a thorough understanding of the research topic and its relevance to health disparity and health equity through iterative communication and formal tracking of activities with the TL 1 research scholar, (3) actively participating during the presentation of the research topic by the TL 1 research scholar at the C-RAC meeting, (4) presenting his/her understanding of the research topic, personal experience, and implications for community benefits of the presented research, and (5) reflecting again to demonstrate understanding and evaluate the extent to which suggestions were implemented by the TL 1 scholar who illustrates on the research results at a 4–6 month follow-up session. Teach-Back sessions led by community members of C-RAC are now implemented in programs where established and aspiring researchers formally share information with the community.

To acquire a better understanding of equity in healthcare research, community partners took an initiative to increase their knowledge about the regulatory definitions and types of research that involve human subjects as participants. This community-initiated effort culminated in 12 community partners getting certified with the human subject research training (online) provided by the U.S. Office of the Human Research Protections

The CAP around equity issues made both sides realize that there was a strong need to improve the health literacy of community around research topics presented by the academic partners. Efforts are on the way from researchers to design and implement more innovative ways to communicate their research with the community.

### Inclusion

One metric of successful DEI is the increase in community representation provided by community partners on academic forums that needed community input and active participation toward a shared CAP agenda. These include community representation at the Johns Hopkins ICTR Leadership Council, National Institutes of Health (NIH) Community Engagement Alliance (CEAL) for the District of Columbia, Maryland, and Virginia Executive Committee, and the NIH CEAL Team Publication Committee.

A critical tenet of these committees is to develop and practice initiatives that incorporate community voices into their agenda. All of the above committees required involvement in activities requiring substantive time during the workday. The community representatives were given herein the opportunity to provide community voices, give feedback on issues that impact the community, and subsequently discuss these issues at the C-RAC meetings.

With these meaningful inputs and engagement throughout the entire research process, the CAP demonstrated that community input has the potential to impact funding, recruitment and retention, research participant informed consent, quality of educational materials, and dissemination of research.

### Evidence of DEI in Successful Community-Engaged Research

Albeit brief, this special communication illustrates several DEI strategies toward successful and continued community-engaged research that benefits community partners directly or indirectly. Most of these strategies are initiated, implemented, and routinely monitored for quality improvement purposes, mostly by community partners. Thus far, incorporation of community inputs using DEI strategies in academic research has resulted in eleven grant proposals submitted to the National Institutes of Health and the Patient-Centered Outcomes Research Institute. Six of these applications have been awarded funds for community-based participatory research. The CAP has also contributed to twelve research publications. Table 1 shows data supporting program successes in dissemination of research findings to academic and community audiences. In addition to the C-RAC membership composition to demonstrate CAP diversity, providing research-based information to diverse consumer groups in local community markets has also strengthened the CAP. Community-initiated annual conferences (Healthy Aging), outreach activities (Day At The Market), formal feedback reflecting community concerns, as well as shared governance in the C-RAC organizational structure also reflect a growing equitable distribution of shared responsibility (Table 1). Conversely, inclusion of community partners in the decision-making process throughout the research continuum, and providing research-related training, although nascent, demonstrate the extent of inclusivity in the CAP.

**Table 1.** Outcomes of community-academic partnership (CAP) activities to ensure DEI

Categories		Partnership activities	Outcomes
**1**	**D**	C-RAC Membership	Thirty-two members from diverse groups
**2**	**D**	Community in Action: Day At The Market	Research awareness across diverse consumer groups in local community markets
**3**	**E**	Research Consults by Community Partners	Guidance/advise to researchers throughout the research continuum
**4**	**E**	Community-initiated outreach activities	Annual programs inviting researchers to provide cutting-edge information on prevention and treatment of clinical topics of community interest and medical ethics
**5**	**E**	Community-initiated outreach activities	Clinicians and researchers invited by community members at local markets to discuss medical research topics of interest to community
**6**	**E**	Shared Governance	Governance committee includes community and academic partners with an elected chair from community
**7**	**E**	Response to NIH-RFI on DEI	Community-initiated reporting and communications – April 2021
**8**	**I**	Nuts & Bolts in Community-Engaged Research	Expanded awareness and knowledge on community-engaged research – three workshops
**9**	**I**	Henerietta Lacks Annual Forum	Expanded awareness and knowledge on DEI
**10**	**I**	Healthy Aging Annual Forum	Expanded knowledge on Aging and Health > 50 topics
**11**	**I**	Functions of the IRB	Expanded knowledge and training on what is IRB and how it works – two trainings and workshops
**12**	**I**	Research Manuscript Writing and Authorship	Collaborative manuscript writing skills training
**13**	**I**	Recruitment and Retention of Human Subject Research Participants	Community consults on recruitment and retention of research participants
**14**	**I**	CTSA – TL 1 Scholars Research Feedback	Community partners Teach-Back on research queries>50
**Overall DEI**		CAP with DEI in Research Continuum	Six grant awards, 12 publications, 5 academic committee membership

Table 1 illustrates that the overall CAP activities embrace a DEI paradigm with overlapping activities. Overall, successful DEI in the CAP is clearly reflected by the funding awards, journal publications, and committee membership as part of a growing relationship.

To promote equity in the community-academic partnership, issues that impact the community are initiated by community partners at scheduled meetings of the C-RAC. In these interactive community grand round forums, invited community leaders present details of current health issues that affect their communities. On different occasions and upon invitation by community partners, academic partners present their research that impacts the community’s health. In both instances, feedback and suggestions from community members are noted for incorporating them into future research practices. The ongoing annual Healthy Aging Forum has been a community-initiated forum that has in recent years incorporated topics such as “sister circle” and “man cave” to discuss cutting-edge research-based applications on men’s and women’s health, respectively. These small group, open-ended, interactive sessions have been popular by overwhelming demands from the lay community in the past five years. The C-RAC is currently in the preparatory process of a Handbook of Community Engagement in Healthcare in this regard.

### Benefits for Community Partners

The current structure in the CAP has been, to a large extent, focused on community consults and is initiated by academic research partners. However, in recent years, the C-RAC has been developing mechanisms for intellectual benefits to community partners. Tangible and intangible benefits received by community partners include: (1) being invited, and compensated as subsequent research team member, (2) sharing governance of the ICTR and C-RAC, (3) getting training and certifications on human subject research, (4) initiating manuscript writing processes (including this report) for peer-reviewed journals, (5) membership in national and regional community advisory boards e.g. CEAL and Community Connect, and (6) receiving financial compensation for attending C-RAC meetings.

### Benefits for Researchers

CAP benefits received by academic partners include: (1) successful applications for federal, state, local, public, and private funding opportunities, (2) improved recruitment and retention of research participants, (3) streamlining of informed consent and IRB applications, (4) publications in peer-reviewed journals, acknowledging contributions from community partners.

Measuring the researcher’s ability to conduct successful community-engaged research also included a recent semi-structured survey among researchers on whether the C-RAC consultation improved the quality of their research, and whether the community consultations provided feedback on proposal development, informed consent, recruitment and retention, and dissemination. More than two-thirds reported positively on the benefits received from community consultations and inputs on participant recruitment and retention. Overall, investigators who utilized the consultation service reported a wide range of positive impacts on research projects, including ability to apply for funding, and development of long-term partnerships.

### Example of Co-Learning Opportunities in CAP

**Figure 3.**
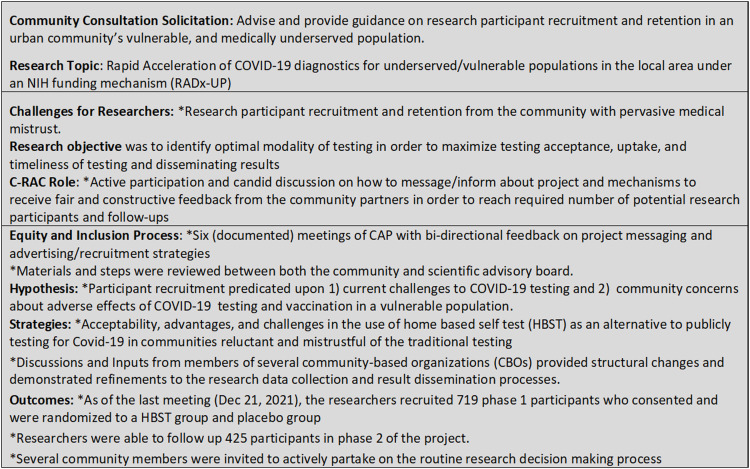
Co-learning opportunities in CAP and successful DEI.

Figure 3 illustrates evidence of successful CAP when utilizing the DEI paradigm. Researchers reported increased understanding of community views on research and health priorities of the community toward a successful community-engaged research. Community members stated that participating in consultations gave them an opportunity to educate researchers about the concerns of people living in their community and reported increased understanding of the research process. Figure 3 illustrates evidence of equity and inclusion *via* active participation, iterative discussions, strategic actions, and improved trust among the partners. Additionally, a fifty-nine percent (425 out of 719) follow-up in the project demonstrates a successful CAP resulting in participant retention when the principles of DEI are applied.

## Discussion

This report describes steps implemented by JH-ICTR C-RAC to achieve DEI *via* successful CAP. The C-RAC adheres to the framework shown in Fig. 1. The commitment to listening, understanding, improving trust, and reciprocating community voices (“give back”) has resulted in dynamic DEI engagement and innovative strategies initiated by community partners and supported by academic partners. C-RAC activities that are responsive to roadblocks *via* DEI strategies are shown in Table 2.

**Table 2.** Community-Research Advisory Council (C-RAC) activities that are responsive to roadblocks *via* DEI strategies

CTSA framework for DEI	Responsive C-RAC activities toward a successful community-academic partnership
Addressing fear of research intention and negative perception of research	• Promoting open-mindedness, consideration of different styles of communication/ health literacy, and showing respect when listening without disagreeing • Providing feedback to community queries by clarifying and confirming what is communicated to ensure community concerns are accurately documented and followed-up • Sharing research information that is not normally included/addressed (e.g. patient’s bill of rights, fair practices/treatments, the Hippocratic Oath) and community implications of research • Demonstrating accountability, transparency, and a time schedule for reporting back reliable results/research outcomes
Alleviating mistrust in research	• Facilitating bidirectional communications to understand the rationale, value, and benefit of the proposed research, and developing collective work and shared responsibility toward sustainable solution to community concerns
Translating research to practice	• Developing tools, guidelines, and practices that are realistic, easily understood, and disseminated to the greater community • Conducting pilot programs with agencies within the community • Providing ongoing recruitment, comprehensive training, and monitoring of Community Health Advocates.
BiDirectional Community-Engaged Research	• Increasing the level of community engagement through outreach, consult, participatory research communication, collaboration, and shared leadership

### Unique Strategies in Implementing DEI

In this report, we have noted a number of unique and intended DEI strategies, which have been beneficial to both the community and academic partners.

The C-RAC is reflective of many unique characteristics that are not visible, at least in similar literature, and interactions with other CTSA awardees in the nation. For example, as part of the enriched consultations, the academic partners from the four independent divisions (Medicine, Nursing, Public Health, and the Hospital) continuously engage with the community partners on a weekly basis toward the monitoring and improvement of a community-engaged research from the beginning of a grant solicitation application and throughout the research continuum process.

A second unique partnership effort as part of Hopkins Opportunity for Participant Engagement initiated by the C-RAC in the past three years has resulted to date in recruitment with consent, of more than seven thousand research participant volunteers who are then triaged to disease/treatment specific researcher/provider specialist.

A third unique and notable partnership characteristic has been the recent linkage between academic cardiovascular research partners and C-RAC community partners who are engaged in substance abuse and mental illness treatment and recovery services for vulnerable population such as drug addicts and the homeless in urban settings. Such partnership not only provides promising practices to study and offers cardiovascular (Linked BP and Linked Heart Studies) services for substance abusers but also focuses both on the limitations and potential of unique and successful community-engaged research that improve health outcomes among vulnerable, urban populations.

Three general themes in the broader CAP framework emerged when community partners were asked to suggest ways of improving DEI: (1) Ensuring that community members of diverse backgrounds are involved and well-informed about the research continuum process, terms, importance, and value of their input through advance preparation of community-engaged research activities with simple, understandable, and clear communication format; (2) incorporating bidirectional feedback from the community members and researchers throughout the process (“consistent feedback of what input and advice from the community has been used”): all researchers were required to send a completed report of their project findings that commensurate with community feedback, and lastly, (3) marketing of the community consultation services to ensure that the community advice benefits future researcher with similar domains/specialties.

As presented earlier, the community partners' initiation of the Community Grand Rounds demonstrates equity wherein invited community leaders have discussed issues of structural racism and discrimination, white supremacy, and unmet health services/research needs in their respective communities and what is important to them from research perspective.

Community partners have expressed interest in an array of activities relevant to “inclusion“ including receiving training/workshops about topics from researchers so as to improve: (1) disseminating research results to study participants and the community, (2) learning about different research methods, (3) preparing community driven grant proposals, (4) successful and sustainable strategies to maintain research partnerships or programs that become critical to community-engaged research projects, (5) developing a research question, (6) best ways to recruit and keep research participants in studies, and (7) the nuts and bolts underlying research ethics including informed consent.

### Lessons Learned/Current Challenges

The community-academic partnership, albeit a successful venture, frequently faces challenges that need to be efficiently addressed toward improving mutual trust in research. For example, learning the dynamics of a complex research process, from definition, methodology, conduct, and dissemination as explained by seasoned researchers, has been a challenging learning process for the nonacademic community partners. On the other hand, academic researchers, while grappling with the cumbersome tasks of recruiting hard-to-reach, mistrusting, vulnerable, eligible research participants, have to rely heavily on community partners to learn successful recruitment and retention strategies. Rather than budgeting reasonable resources to recruit hard-to-reach eligible participants, the perception that participant recruitment needs the least resources may need serious reconsideration.

The CAP frequently faces two other critical challenges that are related to successful research: (1) transparency and disclosure of tangible benefits that the research would bring to community where the research is being conducted, and (2) dissemination of research findings to the same community who participated in the research. Current research protocols, including those submitted to IRBs, only abbreviate these activities but do not expand them, which could improve research participation.

As a continuously evolving process, the partnership sets forth quarterly meetings to introspect critical activities toward strengthening the partnership. Issues that community partners have raised are (1) use of budget lines to formally include interested community partners in grant proposals with post-funding roles and responsibilities, (2) developing mechanisms for community partners to actively participate in the research decision-making process, (3) allocating time and space for community partners to discuss partnership governance, administration, and financial and technical support issues, (4) expediting the compensation process, (5) evaluating the power dynamics of the CAP related to the structural hierarchy in academic settings, (6) tapping into community talent while respecting community time, and (7) recruiting younger generation (e.g. less than 30 years of age) as community partners to learn the dynamics of a CAP. Each of these challenges is currently being addressed as joint CAP endeavors.

### Sustainability

How to sustain an evolving CAP has been a continuing challenge among both community and academic partners, particularly on resource allocation and time commitment toward bidirectional communication in achieving desired CAP impact. Currently, community partners are working toward generating resources by tapping into public or private funds that are available to community-based organizations that address structural racism and discrimination and improve health outcomes.

### Future Direction to Measure Effectiveness of DEI

To an extent, the CAP works in synergy and collaboratively by designing and implementing practical training and exercises to reinforce DEI efforts in the community-partnered research decision-making process. By continuously refining the logic model, the community partners will be able to understand the structure, process, and outcome of rigorous research by the academic partners, and learn that each research follows specific steps encompassing the research continuum that can be measured and documented.

Regardless of whether a research problem or issue is initially identified by either community or academic partners, a fully engaged CAP will need, from the very onset, to jointly develop a research question, formulate a hypothesis, and design the methodology, data analysis, and plan to disseminate results. Such opportunities will provide the optimal environment for community partners’ appreciation of the intricate research process, and in reciprocation, enable the academic partners to build on the partnership to identify innovative ways to improve the community-engaged, bidirectional research [29,30].

In summary, the CAP effectively utilizes the C-RAC to provide (1) expanded knowledge towards creating a bidirectional learning community, (2) shared governance with built-in trust, (3) a sustainable diverse membership, and (4) community-aligned solutions in a diverse, equitable, and inclusive environment.
